# Tamoxifen-independent recombination of reporter genes limits lineage tracing and mosaic analysis using CreER^T2^ lines

**DOI:** 10.1007/s11248-019-00177-8

**Published:** 2019-10-22

**Authors:** A. Álvarez-Aznar, I. Martínez-Corral, N. Daubel, C. Betsholtz, T. Mäkinen, K. Gaengel

**Affiliations:** 1 Department of Immunology, Genetics and Pathology, Rudbeck Laboratory, Uppsala University, Dag Hammarskjöldsväg 20, 75185 Uppsala, Sweden; 2 Integrated Cardio Metabolic Centre (ICMC), Department of Medicine Huddinge, Karolinska Institutet, Novum, Blickagången 6, 141 57 Huddinge, Sweden

**Keywords:** CreER^T2^, Cre/loxP system, Lineage tracing, Mosaic analysis, Tamoxifen-independent recombination, Reporter-gene

## Abstract

The CreER^T2^/loxP system is widely used to induce conditional gene deletion in mice. One of the main advantages of the system is that Cre-mediated recombination can be controlled in time through Tamoxifen administration. This has allowed researchers to study the function of embryonic lethal genes at later developmental timepoints. In addition, CreER^T2^ mouse lines are commonly used in combination with reporter genes for lineage tracing and mosaic analysis. In order for these experiments to be reliable, it is crucial that the cell labeling approach only marks the desired cell population and their progeny, as unfaithful expression of reporter genes in other cell types or even unintended labeling of the correct cell population at an undesired time point could lead to wrong conclusions. Here we report that all CreER^T2^ mouse lines that we have studied exhibit a certain degree of Tamoxifen-independent, basal, Cre activity. Using Ai14 and Ai3, two commonly used fluorescent reporter genes, we show that those basal Cre activity levels are sufficient to label a significant amount of cells in a variety of tissues during embryogenesis, postnatal development and adulthood. This unintended labelling of cells imposes a serious problem for lineage tracing and mosaic analysis experiments. Importantly, however, we find that reporter constructs differ greatly in their susceptibility to basal CreER^T2^ activity. While Ai14 and Ai3 easily recombine under basal CreER^T2^ activity levels, mTmG and R26R-EYFP rarely become activated under these conditions and are therefore better suited for cell tracking experiments.

**Electronic supplementary material:**

The online version of this article (10.1007/s11248-019-00177-8) contains supplementary material, which is available to authorized users.

## Introduction

Genetic studies in mice have revolutionized biological research and have been fundamental to develop animal models for the study of human diseases. Although there are obvious differences between mice and humans (Perlman 2016), mouse-based animal models are often the first choice and allow us to investigate the mechanisms of disease initiation and progression in great detail (Nguyen and Xu 2008). A combination of factors places the mouse at the forefront as a genetic model organism. The mouse is closer related to humans than other frequently used non-mammalian model organisms such as worms and flies and thus far better suited to investigate the complex physiological systems that mammals share. Moreover, the mouse genome is completely sequenced (Mouse Genome Sequencing Consortium et al. 2002) and an ever-expanding repertoire of molecular and genetic techniques allow sophisticated experimental design.

A breakthrough for the mouse as a model system came with the introduction of techniques that allowed the creation of knock-in and knock-out mice. The ability to activate or inactivate a gene of interest has allowed scientists to experimentally test hypotheses regarding the function of specific genes and to determine their role during development, physiology and in pathological settings. While we will continue to learn from conventional knock out techniques, the approach has its shortcomings. The global deletion of genes that are essential for embryonic development is often only partially informative. While the function of such genes can be studied in the embryo, it is often difficult to distinguish primary from secondary effects and it remains unknown if such genes have roles later in development or in homeostasis (Turgeon and Meloche 2009).

**Fig. 1:**
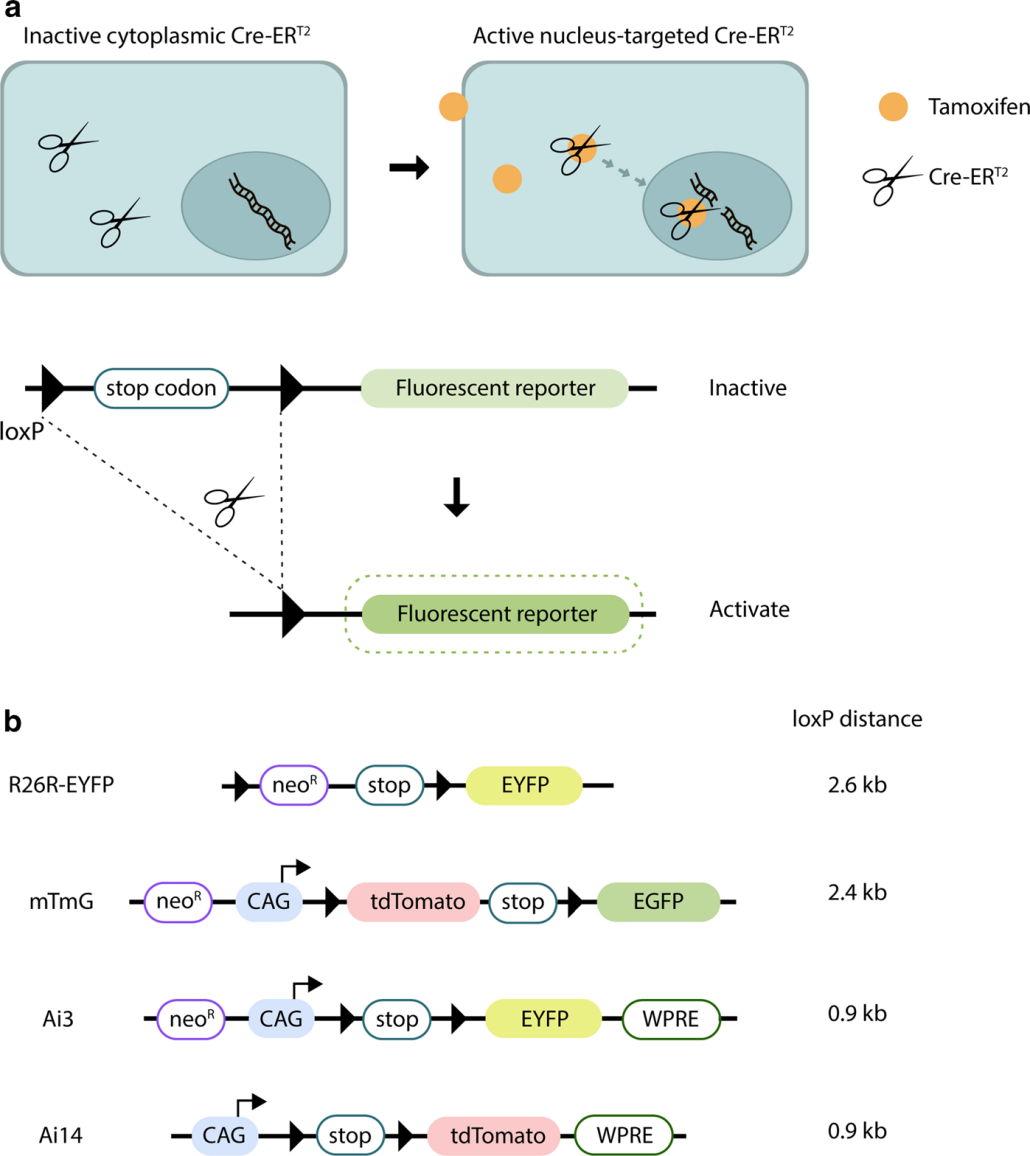
Principle of the CreER^T2^/loxP system and schematic representation of commonly used fluorescent reporter constructs. **a** In the absence of Tamoxifen, CreER^T2^ cannot efficiently enter the nucleus and thus will not recombine target genes. Tamoxifen administration causes CreER^T2^ translocation to the nucleus and results in target gene recombination. In the case of the depicted reporter, excision of the loxP flanked stop codon leads to expression of the downstream-located fluorescent reporter gene. **b** Schematic representation of R26R-EYFP, mTmG, Ai13 and Ai14 reporter lines. LoxP sequences are depicted by triangles. neo^R^: neomycin resistance, stop: stop codon, CAG: CAG promoter, WPRE (posttranscriptional regulatory element). The distance in between the loxP sites is indicated for each line (in kb)

Limitations of traditional global knock-out approaches can be overcome with conditional knock-out techniques. The most commonly used approach is based on the Cre/loxP system (Sauer 1998; Hall et al. 2009). It consists of the site-specific DNA recombinase Cre, encoded by the *cyclizationrecombination* gene of bacteriophage P1, and a 34-bp DNA target sequence, the *loxP* (locus of X-over of P1) site. The loxP site spans two 13 bp inverted repeats that flank an 8-bp non-palindromic central region, which gives the site directionality. Cre efficiently recombines DNA sequences in between pairs of loxP sites (Hoess et al. 1990). Depending on the orientation of the flanking loxP sites, this results in either the excision or inversion of intervening DNA segments. In order to conditionally delete a gene of interest, transgenic mice need to be created in which the DNA sequence to be deleted is flanked by loxP sites. In the absence of Cre, the targeted gene will remain intact. Conditional gene deletion is achieved by placing Cre recombinase under the control of a tissue- or cell type-specific promoter and combining both constructs in the same animal (Schnütgen et al. 2003). Additionally, Cre/loxP allows the inducible overexpression of transgenes or reporters. This requires a transgenic mouse in which the gene of interest is downstream of a loxP-flanked (hereafter referred to as floxed) STOP codon. Cre mediated excision of the STOP codon subsequently results in expression of the desired gene or reporter (Abe and Fujimori 2013). While the above approach allows tissue- or cell type- specific gene deletion or over-expression, it does not permit a temporal control beyond the choice of promoter. This problem can be overcome when Cre is fused to a modified estrogen receptor (CreER), which prevents Cre from entering the nucleus (Feil et al. 1996). When Tamoxifen, an estrogen receptor agonist, is administered, it binds to the receptor and elicits the translocation of CreER to the nucleus where it can recombine the floxed target sites (Fig. 1a). The possibility to control gene expression or deletion in time has made the CreER enormously popular. It has been widely used to study the function of early lethal genes at later developmental stages or in adulthood. In combination with reporter genes, the CreER system is also commonly used for lineage tracing and pulse-chase experiments (Jensen and Dymecki 2014; Kretzschmar and Watt 2012). It has further been utilized to study the behavior or distribution of knock out cells in mosaic tissues (Laviña et al. 2018; Zhang et al. 2018). All these cell-tracking experiments rely on reporter genes that become activated upon Cre mediated recombination in target cells. The most commonly used reporter lines are based on fluorescent proteins whose expression depends on Cre-mediated recombination. Fluorescent proteins are available in a wide absorption–emission range and vary in brightness and photostability (Shaner et al. 2005). In order for lineage tracing to be reliable, it is important that the cell labeling approach only mark the desired cell population and their progeny. Unfaithful expression of reporter genes in other cell types or unintended labeling of even the correct cell population at an undesired time point can lead to wrong conclusions. First generation-CreER mouse lines suffered from this problem and were leaky to various extents (meaning Cre entered into the nucleus in the absence of Tamoxifen, resulting in background activity). In order to limit this background Cre activity and improve the response to Tamoxifen, CreER^T2^, a modified version of CreER, was developed (Feil et al. 1997). While generally less leaky and more reliable than the original CreER lines, background recombination independent of Tamoxifen has also been reported for some CreER^T2^ lines (Kristianto et al. 2017; Fonseca et al. 2017), and differences between some reporters have been noted (Liu et al. 2013). Nevertheless, the potential extent of this phenomenon compels to conduct systematic investigations. In this study, we analyzed three CreER^T2^ and four floxed fluorescent reporter lines, and we discuss their potential pitfalls. We found that all tested transgenic CreER^T2^ lines are leaky to a certain extent and that the analyzed reporter lines have vastly different recombination thresholds. Our data indicate that tracking experiments in which CreER^T2^ lines are used in combination with easily recombining floxed reporters should be interpreted with caution, and that the inclusion of non-Tamoxifen-induced control animals that carry both the CreER^T2^ and the reporter allele, is a necessity for such studies.

## Results

### Reporter gene expression in the absence of Tamoxifen induction reveals inherent background recombination of the CreER^T2^/loxP system

**Fig. 2:**
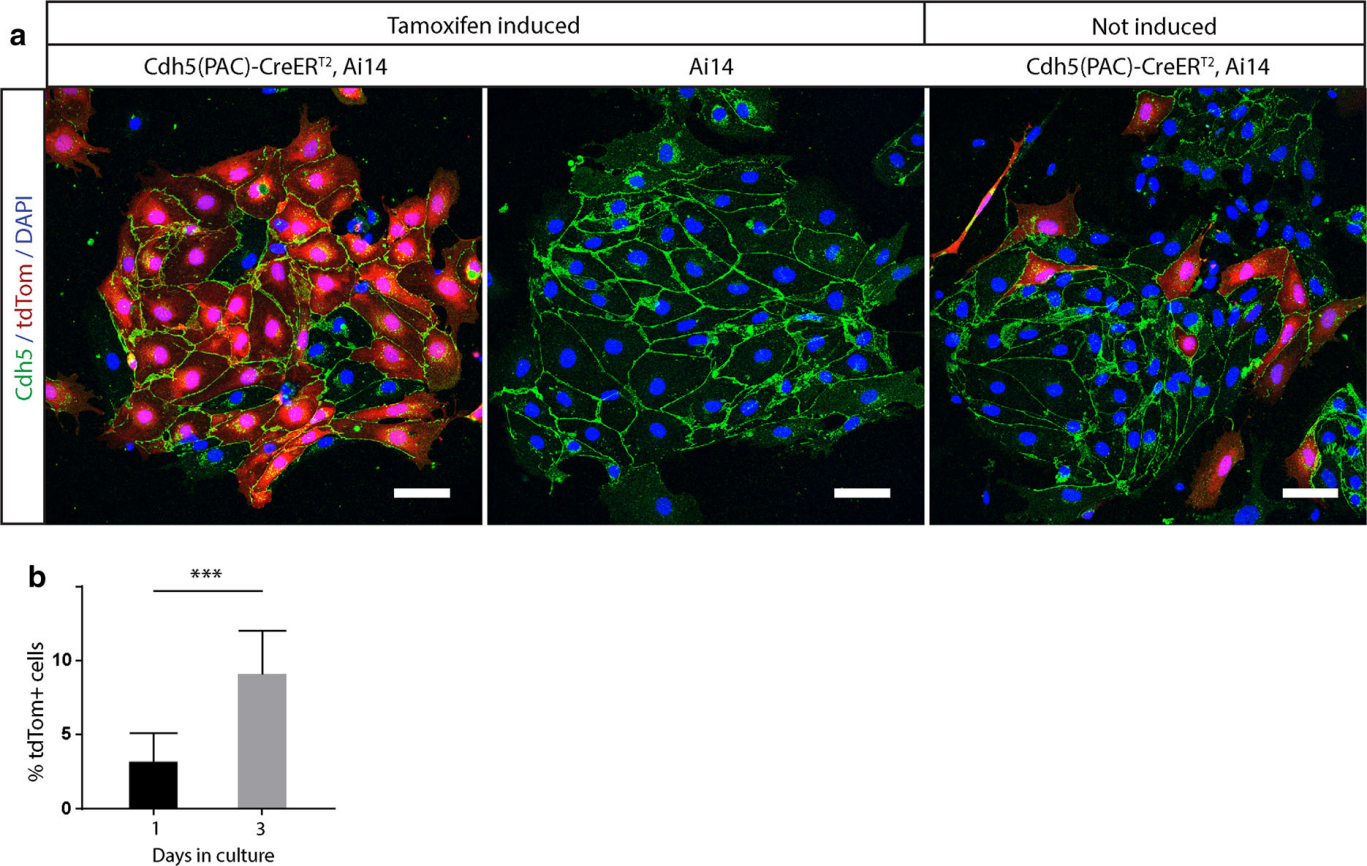
Tamoxifen-independent activation of the Ai14 reporter in primary brain EC isolated from Cdh5(PAC)CreER^T2^ mice. **a** Confocal images of primary brain endothelial cells. Left panel: clusters of EC isolated from Tamoxifen-induced Cdh5(PAC)Cre-ER^T2^/Ai14 animals exhibiting a high percentage of tdTomato expressing cells. Middle panel: EC clusters from Tamoxifen-induced mice carrying only the Ai14, but not the Cdh5(PAC)-CreERT2 construct do not show tdTomato expression. Right panel: endothelial clusters from animals that were not Tamoxifen induced but carry both Cdh5(PAC)-CreERT2 and Ai14 show several tdTomato positive cells and reveal a low level of basal Cre activity. Scale bars: 75 μm. **b** Quantification of the percentage of tdTomato positive cells in cultures of non-Tamoxifen-induced Cdh5(PAC)Cre-ERT2/Ai14 animals one and three days after isolation. ***P ≤ 0.001

We first noticed Tamoxifen-independent, CreER^T2^-mediated activation of a reporter gene when isolating endothelial cells (EC) from mouse brains as an experimental control. We had combined the endothelial specific Cdh5(PAC)-CreER^T2^ line (Pitulescu et al. 2010), our gene of interest and the widely used Ai14 reporter (Madisen et al. 2009) and subjected those mice to various tamoxifen induction regimes. In order to estimate the recombination efficiency we subsequently isolated primary EC from Tamoxifen-induced animals and used non-induced littermates of the same genotype as experimental controls. As expected, EC from Tamoxifen-treated mice showed widespread expression of tdTomato, the fluorescent protein of the Ai14 reporter. However, surprisingly, we also detected a small number of tdTomato positive cells in the EC culture that were isolated from non-induced control littermates. To eliminate any confounding factors that the floxed gene present in the original study could impose, we repeated the experiment using mice that only carried the Cdh5(PAC)-CreER^T2^ and one allele of the Ai14 reporter. We induced a portion of those mice with Tamoxifen and subsequently isolated EC and compared them to EC isolated from control mice that had never been exposed to Tamoxifen. We observed a high degree of tdTomato positive cells in cultures from Tamoxifen-induced mice and again, a small portion of tdTomato positive cells in cultures from non-induced mice. Importantly, we never observed tdTomato positive cells from Tamoxifen-induced mice that carried the Ai14 reporter but not the Cdh5(PAC)-CreER^T2^ allele (Fig. 2a). This suggests that tdTomato expression is not the result of Cre-independent spontaneous recombination of the Ai14 reporter, but rather the result of low level Cre-background activity from the Cdh5(PAC)-CreER^T2^ line. The fact that we observed tdTomato positive EC already during the early steps of the isolation procedure, before the cells even adhered to the cell culture plate (data not shown), strongly suggests that reporter activation had already occurred in the animal and was not induced by the isolation procedure itself. Interestingly, we also noticed that the percentage of tdTomato positive cells from non-induced Cdh5(PAC)-CreER^T2^, Ai14 mice increased with time in culture. After two days tdTomato positive cells accounted for 8,87% (s.d: 0,41) of EC, compared to 3,61% (s.d: 1,7) on the day of isolation (Fig. 2b). We do not know for certain what causes the increase of tdTomato positive cells in culture, but a higher demand to establish cell–cell contacts in culture leads to increased Cdh5 (Vascular Endothelial Cadherin, VE-Cadherin) promoter activity (Kiran et al. 2011), and it seems plausible that this could contribute to higher levels of background Cdh5(PAC)-CreER^T2^ activity.

### Fluorescent reporter genes can become activated by background Cre activity in several CreER^T2^ lines

**Fig. 3:**
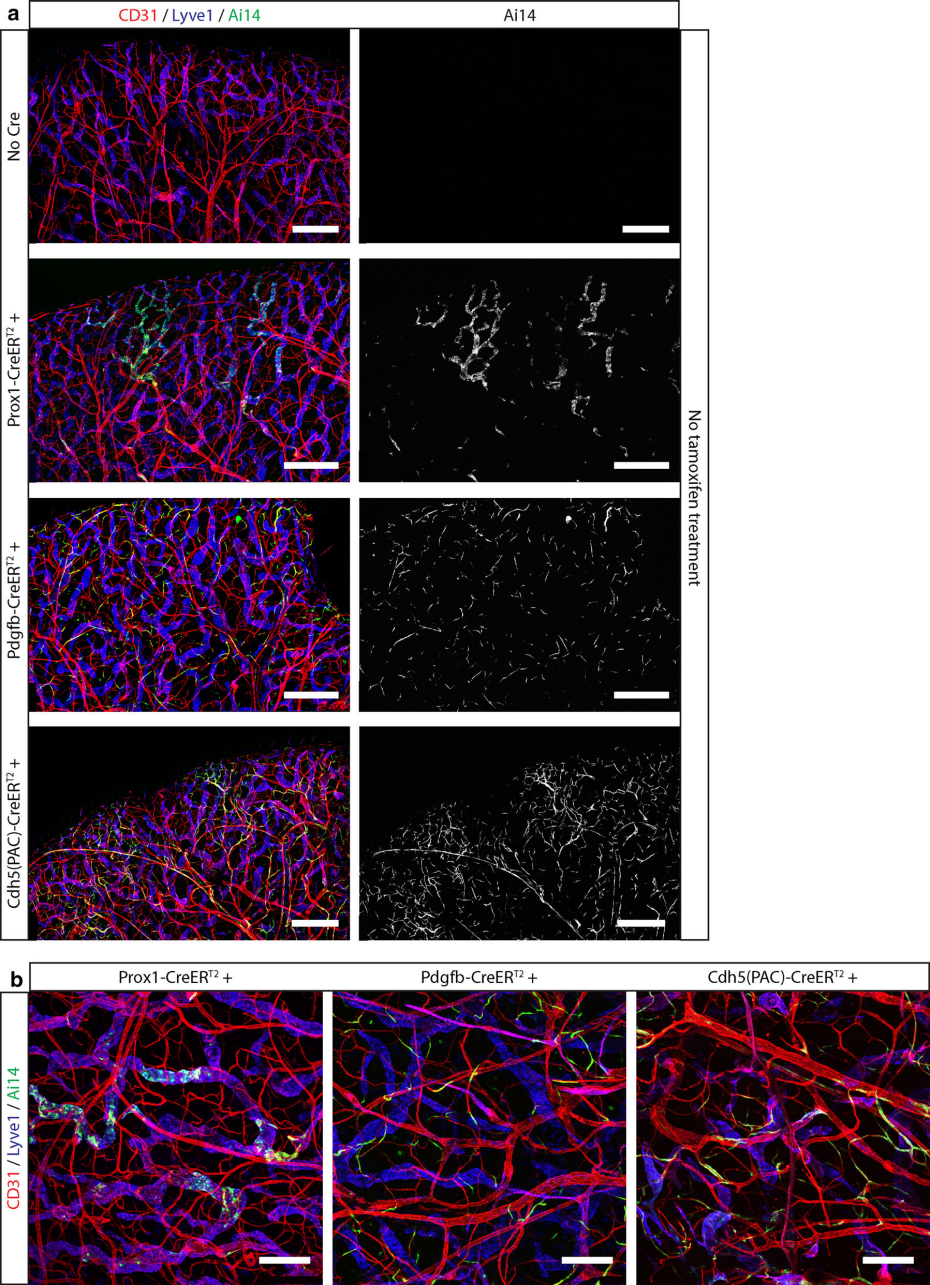
Several CreER^T2^ lines are basally active and lead to reporter gene activation in vivo in the absence of Tamoxifen. **a** Confocal images of the ear skin vasculature of mice carrying the Ai14 reporter alone or in combination with one of the following CreER^T2^ lines: Prox1 (expressed in lymphatic endothelial cells, LEC), Pdgfb (expressed in LEC and blood endothelial cells, BEC) or Cdh5 (expressed in LEC and BEC). Widespread tdTomato expression, a consequence of Tamoxifen independent recombination of the Ai14 reporter, is observed in all CreER^T2^ lines, but it does not occur in the absence of a CreER^T2^ driver in mice that only carry the Ai14 reporter. BEC and LEC are visualized with a CD31 antibody (red) and LEC are stained with a Lyve1 antibody (blue). tdTomato expression from the Ai14 reporter is shown in green. Scale bars indicate 500 μm. **b** Higher resolution images from the pictures in a). Scale bars indicate 200 μm

We next examined if Ai14 reporter activation in non-induced Cdh5(PAC)-CreER^T2^ mice also occurred in vivo. When analyzing the ear skin we observed widespread, tdTomato expression, reinforcing the idea that reporter activation can occur in a Tamoxifen-independent manner (Fig. 3). Importantly however, tdTomato positive cells were only present in endothelial cells—their assumed location—, and we could not detect any recombination in mice carrying the Ai14 reporter alone, suggesting that reporter activation happens in a CreER^T2^-dependent fashion. We subsequently investigated if the Tamoxifen-independent activation of reporter alleles could be observed in other Cre-ER^T2^ lines or if this phenomenon remained restricted to Cdh5(PAC)-CreER^T2^. We tested Pdgfb-CreER^T2^ and Prox1-CreER^T2^ (Claxton et al. 2008; Bazigou et al. 2011) and found that both showed some activation of the Ai14 reporter in the absence of Tamoxifen induction in the ear skin (Fig. 3). Importantly, the fluorescent signal remained restricted to blood and lymphatic vessels in the case of Pdgfb-CreER^T2^ and to lymphatic vessels in the Prox1-CreER^T2^ line. Thus, all tested lines suffered from a basal, low-level, CreER^T2^ background activity or “leakage”, and from subsequent activation of fluorescent reporters in vivo.

### Reporter constructs differ in their susceptibility to basal CreER^T2^ activity

**Fig. 4:**
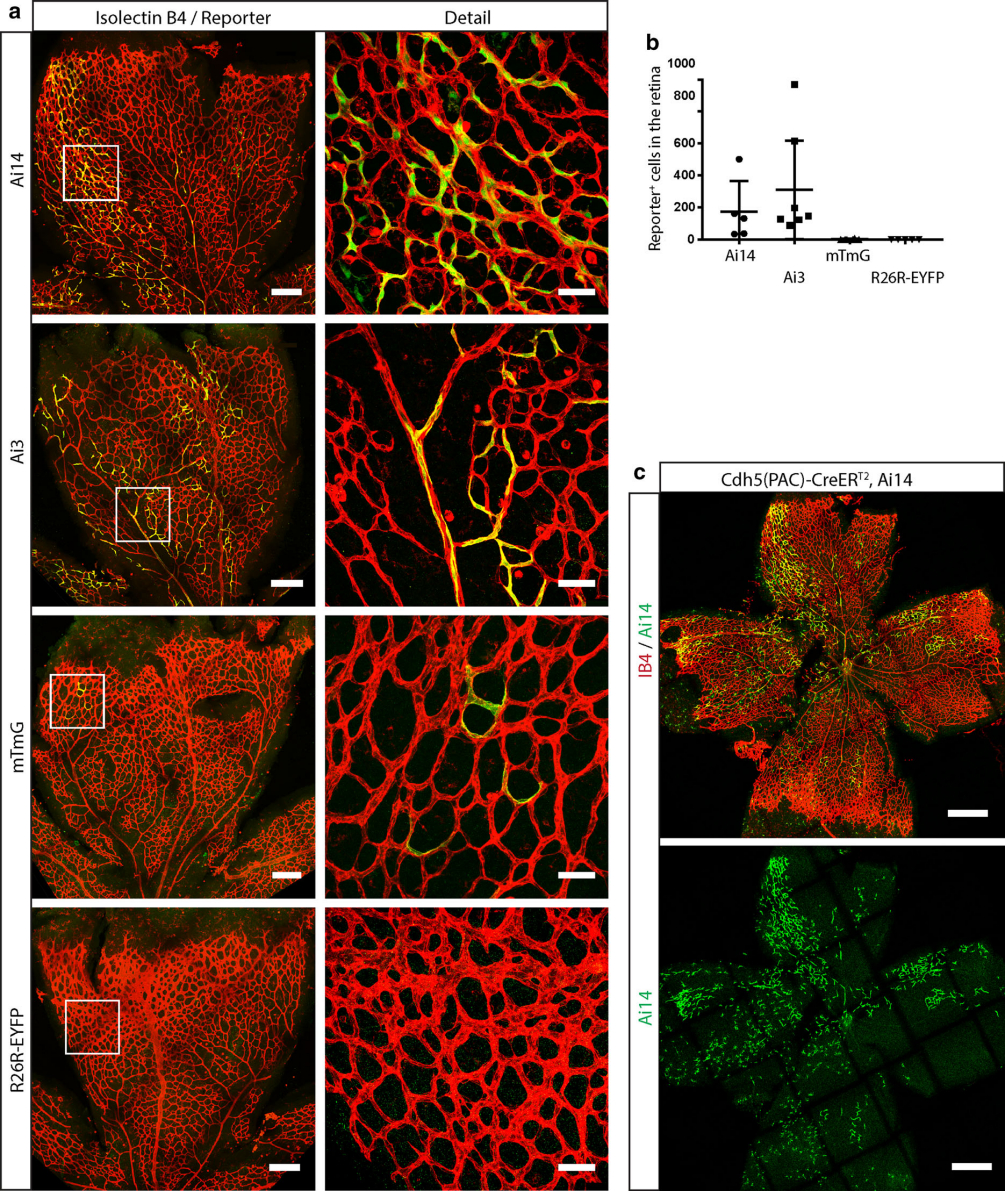
Fluorescent reporters differ in their susceptibility to basal CreER^T2^ activity. **a** Representative confocal images of whole mount retinas from animals carrying Cdh5(PAC)-CreER^T2^ and either Ai14, Ai3, mTmG, or the R26R-EYFP reporter. Endothelial cells are labeled with IB4 (isolectin B4), in red, and fluorescent proteins expressed by the individual reporters are depicted in green. Left column panels: Both the Ai14 and the Ai3 reporters appear to have a low recombination threshold, as basal (non-Tamoxifen induced) Cdh5(PAC)-CreER^T2^ levels are sufficient to induce expression of the fluorescent reporters in a significant amount of cells. In contrast, only few or no fluorescent cells were observed in mTmG or R26R-EYFP retinas, indicating that those reporters recombine inefficiently under basal CreER^T2^ leakage-levels. Scale bars indicate 200 μm. Right column panels: detail of the areas marked with white squares in the left panels. Scale bars indicate 50 μm. **b** Quantification of fluorescent cells found in the retinas of the different reporter mice (Ai14: n = 5, Ai3: n = 7, mTmG: n = 6, R26R-EYFP: n = 5). One way ANOVA was performed for statistical analysis. **c** Extreme example of the degree of reporter activation that can be observed under basal, non-Tamoxifen-induced, conditions using Cdh5(PAC)-CreER^T2^ in combination with Ai14. The vasculature is labeled by IB4 in red, and the Ai14 reporter signal is shown in green. Scale bar indicates 500 μm

**Fig. 5:**
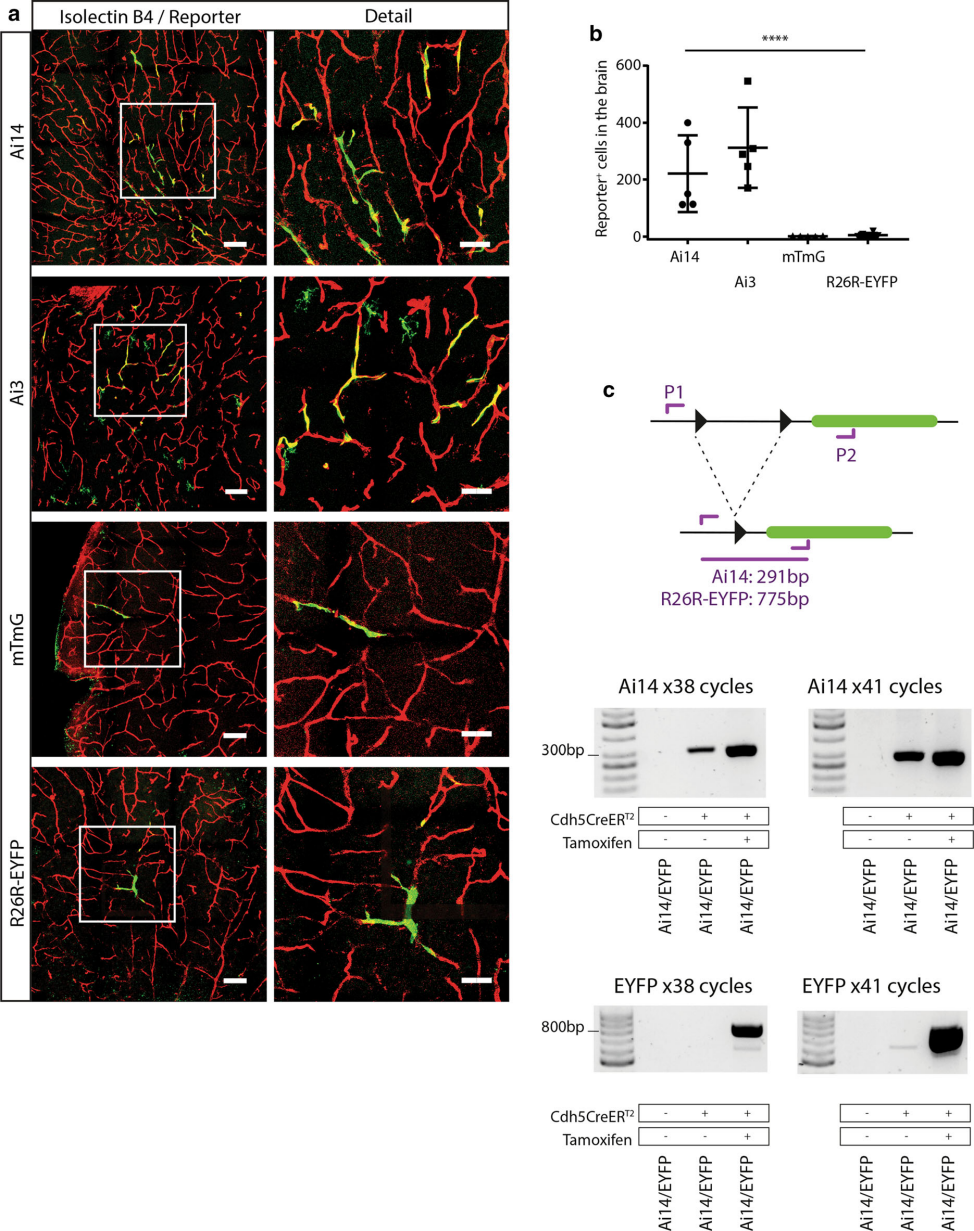
Activation of fluorescent reporters under basal CreER^T2^ activity levels in the brain. **a** Left column panels: representative images of brains sections from Cdh5(PAC)-CreER^T2^ mice with either the Ai14, Ai3, mTmG, or R26R-EYFP reporter. Positive endothelial cells can be seen in all brains to varying degrees. Scale bars indicate 100 μm. Right column panels: detailed images of the areas marked with squares in the left panels. Scale bars indicate 50 μm. **b** Quantification of fluorescent cells in brain sections of the different reporter mice (Ai14: n = 5, Ai3: n = 5, mTmG: n = 5, R26R-EYFP: n = 7). One way ANOVA was performed for statistical analysis. **c** PCR analysis of Cre-mediated recombination in the Ai14 and R26R-EYFP reporter. Two sets of PCR primers (P1 and P2) were designed to flank the loxP sites (indicated by black arrowheads) with P1 priming upstream of the first loxP site and P2 within the fluorescent gene (indicated in green, for Ai14 and R26R-EYFP respectively). PCRs conditions were optimized to amplify the genomic DNA region that remained after recombination had occurred: 291 bp (Ai14) and 775 bp (R26R-EYFP). After 38 cycles, abundant PCR product was obtained for both Ai14 and R26R-EYFP after Tamoxifen induction, while in the absence of Tamoxifen only PCR product from the Ai14 reporter was detected. After 41 cycles, increased amount of PCR product was obtained for both Ai14 and R26R-EYFP after Tamoxifen induction, and in the non-Tamoxifen-treated Ai14 sample, whereas only a very faint band appeared in the non-Tamoxifen-treated R26R-EYFP sample. In the absence of CreER^T2^, no product is detected in any PCR

Next, we investigated if basal CreER^T2^ leakage could lead to the activation of reporter lines other than Ai14. We chose to analyze the commonly used Ai3 (based on Enhanced Yellow Fluorescent Protein, EYFP) (Madisen et al. 2009), R26-mTmG (carrying tdTomato and Enhanced Green Fluorescent Protein, EGFP) (Muzumdar et al. 2007), and R26R-EYFP (based on EYFP) (Srinivas et al. 2001) reporters lines (Fig. 1b). In order to expose those reporters to the same basal level of CreER^T2^ leakage we generated mice that carried Cdh5(PAC)-CreER^T2^ and either an allele of Ai14, Ai3, R26-mTmG, or R26R-EYFP and investigated the level of Tamoxifen-independent reporter gene activation in the retina, brain, and embryonic skin. We first measured the number of fluorescent EC in the retinal vascular plexus at postnatal day (P)7 and found that all Ai14 (n = 5) and Ai3 (n = 7) retinas exhibited an average of over one hundred recombinant cells per retina, suggesting that these reporters are highly sensitive to basal CreER^T2^ leakage (Fig. 4). Interestingly, in the case of mTmG we failed to find reporter positive cells in any but one retina (n = 6), in which we observed less than 10 positive cells. In the case of R26R-EYFP, no analyzed retina (n = 5) presented recombinant cells. Taken together, these results suggest that mTmG and R26R-EYFP have a substantially higher recombination threshold to basal CreER^T2^ leakage than Ai14 and Ai3, which become easily activated (Figs. 4,  7). We obtained similar results when analyzing the brains of these animals. In the case of Ai14 and Ai3 we found on average of 200 to 300 reporter-expressing EC per sagittal brain section, while the amount of reporter positive EC in mTmG and R26R-EYFP brain sections was below ten, or completely negative (Fig. 5). Additionally, in the skin of Cdh5(PAC)-CreER^T2^, Ai14 embryos we found abundant recombinant cells, while we detected very few reporter positive cells in Cdh5(PAC)-CreER^T2^, mTmG embryos (Supplementary Fig. 1). We used male and female mice for our analyses and did not observe an obvious difference in the number of recombinant cells in between the sexes (data not shown).

To corroborate that the divergence in fluorescent cell numbers between reporter lines was the result of recombination events, we designed PCRs to detect the presence of recombined DNA in the Ai14 and R26R-EYFP reporters, respectively (see methods for detail). We generated mice that simultaneously carried the Ai14 and R26R-EYFP reporters, and mice that carried both reporters and Cdh5-CreER^T2^. This approach ensured that both reporters were exposed to the same amounts of basal CreER^T2^ activity. On these mice we subsequently run PCRs to detect recombination events prior to and after tamoxifen induction (Fig. 5c). After 38 PCR cycles, abundant product was obtained for both the Tamoxifen-treated Ai14 and R26R-EYFP samples, while in the absence of Tamoxifen only PCR product for the Ai14 reporter could be detected. To dismiss the possibility that the absence of PCR product in the R26R-EYFP sample was the result of low PCR efficiency we repeated the PCRs with increased numbers of cycles in order to improve the yield. After 41 cycles, the amount of PCR product increased in both the Tamoxifen-treated and un-treated Ai14 sample. In contrast, we only obtained a faint band in the non-Tamoxifen-treated R26R-EYFP sample, despite the fact that the product was strongly amplified in the Tamoxifen-treated sample. In the absence of Cdh5-CreER^T2^, no product was detected in any PCR. The fact that the difference in amount of PCR product between Tamoxifen-treated versus non-treated samples is substantially higher for R26R-EYFP than for Ai14, strongly suggests that Ai14 recombines more easily than R26R-EYFP when exposed to the same degree of basal CreER^T2^ activity.

### Basal CreER^T2^ leakage levels may hamper lineage tracing experiments

**Fig. 6:**
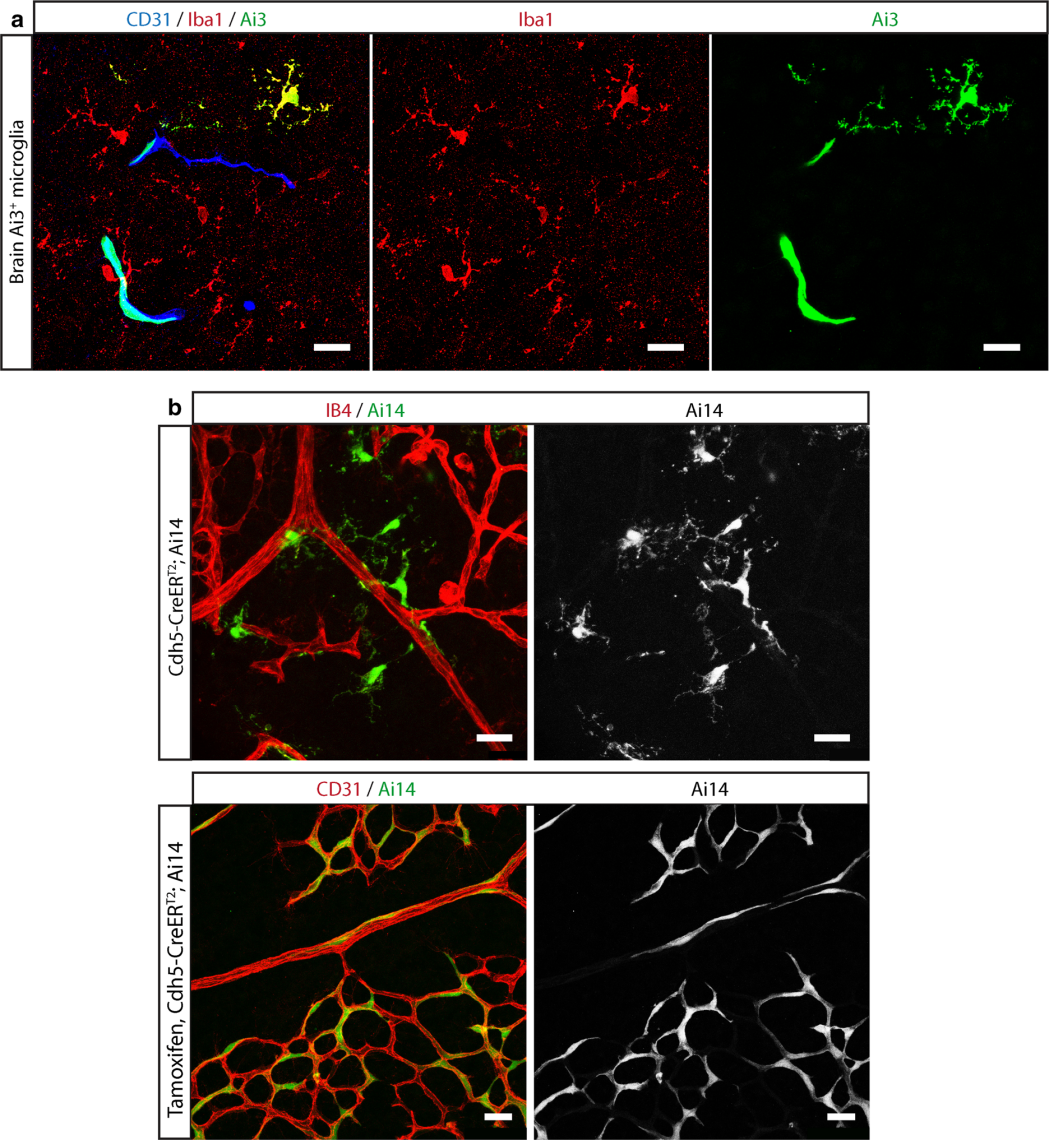
Transient CreER^T2^ expression and leakage in an ancestor cell may hamper lineage tracing in daughter populations. **a** Fluorescent reporter expression in brain endothelial cells and microglia as a result of basal CreER^T2^ leakage in mice carrying Cdh5(PAC)-CreER^T2^ and the Ai3 reporter. Endothelial cells are shown in blue (CD31) and microglia are depicted in red (Iba1). Note that Ai3 (shown in green), is labeling a subset of endothelial cells and microglia. Lower panels represent microglial staining and Ai3 signal, respectively. Scale bars indicate 20 μm. **b** Fluorescent reporter expression in retinas of mice carrying Cdh5(PAC)-CreER^T2^ and the Ai14 reporter. Upper row: reporter expression in microglia due to basal CreER^T2^ leakage. Lower row: representative image of the retina of a Tamoxifen-induced mouse, note the absence of positive microglia in this retina. Vascular outlines are shown in red (isolectin B4 or CD31) and Ai14 reporter expression is shown in green. Scale bars indicate 25 μm

While analyzing the presence of reporter-positive EC in the brain and the retina of non-induced mice carrying Cdh5(PAC)-CreER^T2^ and Ai14 or Ai3, we found that, in a small number of animals, some microglia were also positive for these reporters (Fig. 6). This was surprising, as Cdh5 is a vascular marker, and its promoter is known to drive expression specifically in blood and lymphatic endothelial cells, whereas its expression by mature microglia has not been reported. In addition, single cell sequencing data does not support Cdh5 expression in adult microglia (Vanlandewijck et al. 2018). This suggests that the reporter positive microglia had been labeled earlier in development at a point when the precursors of these microglia must have temporarily expressed Cdh5. This hypothesis is supported by the fact that Tamoxifen-induction of neonatal Cdh5(PAC)-CreER^T2^, Ai14 mice did not result in an increase in the amount of labeled microglia, while it specifically amplified the amount of labeled endothelial cells. In line with this result, several investigations report that Cdh5 is expressed in the early hematopoietic lineage (Fraser et al. 2002; Lancrin et al. 2009). In fact a developmental RNA sequencing database indicates some expression of Cdh5 in embryonic microglia, which is lost later in development (Li et al. 2019).

Taken together, our results show that reporter genes can differ widely in their susceptibility to basal CreER^T2^ leakage and that Ai14 and Ai3 should be used with caution in lineage tracing, pulse-chase or mosaic analysis experiments.

## Methods

### Mouse lines and Tamoxifen administration

Three CreER^T2^ lines were utilized: Cdh5(PAC)-CreER^T2^ (Pitulescu et al. 2010), Pdgfb-CreER^T2^ (Claxton et al. 2008) and Prox1Cre ER^T2^ (Bazigou et al. 2011); as well as four fluorescent reporter lines: Ai14 and Ai3 (Madisen et al. 2009), mTmG (Muzumdar et al. 2007), and R26R-EYFP (Srinivas et al. 2001).

Induction of postnatal mice was performed by oral gavage to the lactating females, with a daily dose of 100 μl 20 mg/ml Tamoxifen (T5648, Sigma-Aldrich) from P0 to P2. Tamoxifen was first diluted in 10% ethanol and further diluted in 90% corn oil (C8267, Sigma-Aldrich).

Induction of adult mice was performed by oral gavage of 100 μl 20 mg/ml Tamoxifen with two doses on alternating days. Both male and female mice were used for our analyses.

### Microvascular fragment isolation, EC culture and immunohistochemistry

Primary endothelial cells from microvascular fragments were isolated and cultured as described before (Niaudet et al. 2015). Briefly, brains from P7 mice were dissected and minced with 1% collagenase (Sigma C6885), 1% penicillin/streptomycin (P/S, Life Technologies 15140-122) DMEM (low glucose, pyruvate; Gibco 31885023) for 20 min while stirring at 300 rpm and 37 °C and neutralized with 20% fetal bovine serum (FBS), 1% PS, DMEM. The suspension was filtered through a 70 µm cell strainer (BD Falcon) and centrifuged at 520 g for 5 min. The cells were resuspended in 1% PS, 0.5 mg/mL heparin (Sigma H3149), DMEM and incubated in rotation at room temperature for 30 min with Dynabeads (Invitrogen) previously coated with rat-anti mouse PECAM1 (BD Pharmingen#553370) antibody. Bead were dissociated with Tryp-LE 10x (Life Technologies). EC were cultured in ECGM2 medium (Promo Cell #C22011) at 37 °C, 5% CO2.

EC in culture were fixed 5 min in 3% PFA (paraformaldehyde) at room temperature and then blocked during 2 h at room temperature in 5% normal donkey serum, 0.2% BSA (bovine serum albumin), 0.1% Triton X-100, PBS (CaCl_2_, MgCl_2_). Cells were incubated overnight at 4 °C with goat anti-VE-Cadherin antibody (Santa Cruz, sc-6458, 1:500) in 0.1% BSA, 0.05 Triton X-100, PBS. After washes in PBS, cells were incubated with donkey anti-goat 488 during 3 h at room temperature, followed by incubations with 0.1 μM 647P-conjugated phalloidin (Promocell, PK-PF647P-7-01) for 30 min, and 1 μg/ml Hoechst (Life Sciences, H3570) for 15 min.

### Retina immunohistochemistry

Eyeballs of P7 or P8 mice were fixed in 4% formaldehyde at 4 °C during 2 h and then washed in PBS and dissected. Retinas were permeabilized and blocked at 4 °C overnight in PBS, 1% BSA, 0.5% Triton X-100, 5% Normal donkey serum (Jackson ImmunoResearch). Ai3 and Ai14 retinas were incubated with isolectin B4 conjugated with Alexa Fluor-647 (IB4, ThermoFisher Scientific I32450, 1:200) in PBlec: PBS, 0,1 mM CaCl_2_, 0,1 mM MgCl_2_, 0,1 mM MnCl_2_, 1% Triton X-100 pH 6.8 for 4 h at room temperature. R26R-EYFP and mTmG retinas were also incubated with chicken anti-GFP (Abcam ab13970, 1:500) during that time. This was followed by three 30′ washes in PBS, 0.5% BSA, 0.25% Triton X-100. R26R-EYFP and mTmG retinas were then incubated overnight at 4 °C with the Donkey anti-chicken 488 (Jackson ImmunoResearch, 703-545-155) in PBS, 0.5% BSA, 0,25% Triton X-100 and washed again three times 30′ in PBS. Retinas were mounted with ProLong Gold Antifade Mountant (Life Technologies, P36930) and imaged on a confocal laser-scanning microscope (Leica TCS SP8) with a 10X magnification objective.

### Brain immunohistochemistry

Brains were fixed in 4% formaldehyde for 4 h at 4 °C upon dissection, and cut in 50 μm thickness sections on a vibratome. Brain sections were blocked and permeabilized at 4 °C overnight in PBS, 1% bovine serum albumin (BSA), 0,5% Triton X-100, 5% Normal donkey serum. Ai3 and Ai14 brain sections were incubated with Alexa Fluor647-conjugated IB4 (1:200) in PBlec during two days at 4 °C. In addition, R26R-EYFP and mTmG were also incubated with chicken anti-GFP. Three washes of one hour were performed in PBS, 0,5% BSA, 0,25% Triton X-100. Afterwards, R26R-EYFP and mTmG brain sections were incubated over two days at 4 °C with Donkey anti-chicken 488 and washed again, while no additional incubation was performed on Ai3 and Ai14 brains. Sections were mounted with ProLong Gold Antifade Mountant and imaged on a confocal laser-scanning microscope (Leica TCS SP8) with a 10X magnification objective.

### PCR analysis of recombination efficiency

The following PCR primers were used to detect the recombined “floxed” R26R-EYFP reporter: P1: CCAGGGTTTCCTTGATGATGTC and P2: GTGGCGGATCTTGAAGTTCAC. These primers anneal upstream and downstream from the floxed site and can amplify two different DNA fragments: a “full length” region including the floxed sequence (3444 base pairs), or a shorter fragment when recombination has occurred (775 bp). The PCR programs for R26R-EYFP consisted of 38 or 41 cycles with 30′’ denaturalization at 94 °C, 45’’ annealing at 53 °C and 45’’ extension at 72 °C. This program favors the generation of the 775 bp band and does not produce a 3444 bp fragment.

For the Ai14 reporter we used: P1: GGTTCGGCTTCTGGCGTGTGACC and P2: AAGGCCGGCCGAATTCGATCTAGC. These primers also bind upstream and downstream from the floxed Ai14 region, and can amplify the full-length region of 1162 bp or a recombined fragment of 291 bp. The PCR programs for Ai14 were as follows: 38 or 41 cycles of 30’’ denaturalization at 94 °C, 45’’ annealing at 60 °C and 30’’ extension at 72 °C. These programs favor the appearance of the recombined band; however, the full-length fragment can also be detected (not shown). To determine the size of the PCR products we used the GeneRuler 50 bp DNA ladder (ThermoFisher Scientific).

### Retina and brain reporter-recombination analysis

The number of cells expressing the fluorescent reporter was measured with the plugin “Analyze particles” on Fiji software, using images of the whole retina or brain to count the number of cells that were reporter-positive. For retinas and brains with a very high recombination, this method was not valid due to the overlap of reporter-positive cells forming a continuum. In these cases, we measured the average size of an individual cell and divided all the reporter-positive area by the size of a cell to estimate the number of recombinant cells.

### Whole-mount immunofluorescence of skin samples

Whole mount tissue (ear skin/embryonic skin) was fixed in 4% paraformaldehyde for 2 h at room temperature, permeabilized in 0.3% Triton X-100 in PBS (PBST) for 10 min and blocked for 2 h in PBST + 3% milk. Samples were then incubated with primary antibodies at 4 °C overnight in blocking buffer, followed by several washes with PBST and incubation with secondary antibodies for 2 h at room temperature. Stained samples were washed and mounted with Mowiol.

The following primary antibodies were used: rat anti-mouse PECAM-1 (553370, BectonDickinson), rat anti-mouse LYVE-1 (103-PA50AG, Reliatech), goat anti-mouse Nrp2 (AF567, R&D Systems) and rabbit anti-GFP (A11122, Thermo Fisher). Secondary antibodies conjugated to Cy3, Alexa Fluor 488 or 647 were obtained from Jackson ImmunoResearch.

## Discussion

The possibility to control the timing of recombination events via Tamoxifen administration has made the CreER^T2^/loxP system enormously popular. Apart from its wide usage in conditional expression or deletion of target genes, it is progressively used in combination with floxed fluorescent reporters for cell tracking experiments such as lineage tracing, or mosaic analysis. The usefulness of the CreER^T2^/loxP system depends on its faithful expression. Any expression in cell populations other than the desired ones or an expression in even the correct cell population at the wrong time can lead to erroneous conclusions. Along those lines, Tamoxifen-independent Cre activity and subsequent activation of reporter genes has previously been reported for individual CreER^T2^ lines (Papoutsi et al. 2015; Kristianto et al. 2017), but it has remained unclear if the reported examples are rare exceptions or represented a general problem. In this study, we analyzed three CreER^T2^ lines: Cdh5(PAC)Cre-ER^T2^, Pdgfb-CreER^T2^, and Prox1-CreER^T2^ and find that all exhibit a certain degree of basal CreER^T2^ activity. Our observations indicate that sequestration of CreER^T2^ to the cytoplasm is not 100% efficient and that leakage of CreER^T2^ into the nucleus occurs stochastically. The frequency of these events seems to correlate with the abundance of CreER^T2^ molecules in the cell. In addition, the amount of reporter activation also seems to depend on the reporter line. Here we compared the four commonly used reporter lines: Ai14, Ai3, mTmG and R26R-EYFP, and find that Ai14 and Ai3 become substantially easier recombined than mTmG and R26R-EYFP. One of the possible reasons for this differential reporter activation could lie in the distance between the loxP sites, which has been shown to have an effect on recombination efficiency (Zheng et al. 2000). The loxP sites flanking the STOP codon in the mTmG and R26R-EYFP reporter constructs are further apart than in the other two lines (Fig. 1b), conferring them a higher recombination threshold. Another factor that may affect recombination is chromatin state, which can alter the accessibility of Cre to the loxP sites (Vooijs et al. 2001).

It could further be hypothesized that differences in promoter strength between the investigated reporters might influence the amount of cells being labeled. Looking at Ai14 and R26R-EYFP this appears, at first sight, a plausible explanation, since Ai14 contains a synthetic CAG promoter, which is substantially stronger than the native Rosa26 promoter of R26R-EYFP (Chen et al. 2011). However, the argument seems less valid in light of the fact that the same CAG promoter is also present in the mTmG reporter, which labels substantially less cells than Ai14.

Differences in the brightness of the fluorescent proteins can neither explain the discrepancies in the amount of labeled cells. While tdTomato is a brighter fluorescent protein than EYFP, equally many cells are labeled by the Ai14 and the Ai3 reporters, yet Ai3 and R26R-EYFP, both of which express EYFP, differ substantially in the amount of cells that become labeled as a consequence of basal CreER^T2^ activity. In addition, throughout this study, EYFP expression was always amplified with an antibody staining which resulted in a uniformly strong EYFP signal in cells that had undergone recombination. Thus, while one might expect that promoter strength or the brightness of the reporter fluorescent protein could have an effect on the amounts of cells being labeled; neither of those factors seems to have a major impact.

In order for lineage tracing to be reliable, it is key that the labeling approach only marks the desired cell population and their progeny. For this reason, CreER^T2^ leakage and subsequent reporter activation events during earlier developmental stages should be taken into consideration. The progeny of cells in which the promoter controlling CreER^T2^ expression was temporarily expressed will inherit the activated reporter. One such example is the labelling of microglia cells in Cdh5(PAC)Cre-ER^T2^, Ai14 mice. Without appropriate controls, this finding could have been erroneously interpreted as a transdifferentiation of microglia from endothelial cells.

For mosaic analysis, the reporter expression must, in addition, be as closely correlated to the gene deletion as possible. In such experiments, reporter expression should be crosschecked with immunohistochemistry to confirm the loss of the gene of interest in the labeled cell population. In cases where reliable immunostaining cannot be achieved, such correlation could be based on a cell-autonomous phenotype (Laviña et al. 2018). However, with the introduction of the ifgMosaic mice (Pontes-Quero et al. 2017), technically superior options now exist. In this approach, gene recombination and reporter activation are fully linked through bicistronic expression.

**Fig. 7:**
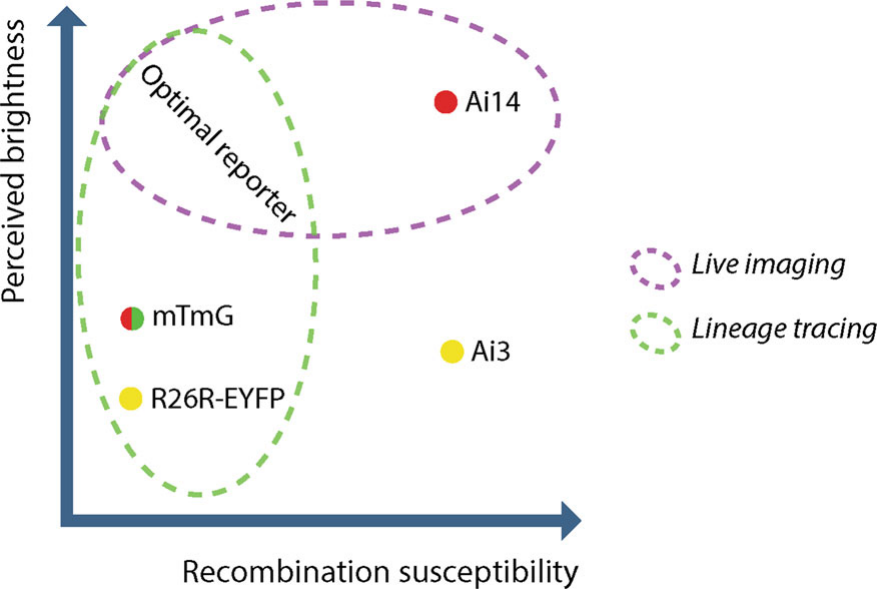
Properties of the tested reporter constructs and recommendations for preferred usage. Diagram depicting brightness differences of the fluorescent proteins in the analyzed reporters as well as their recombination threshold, based on basal CreER^T2^ leakage in non-tamoxifen induced animals. Ai14 is the brightest reporter tested and thus well suited for live cell-imaging approaches. The fact that it easily recombines makes it suitable for experiments in which one aims for a maximum of cells to be labeled. The R26R-EYFP reporter is difficult to image unless amplified by immunohistochemistry and therefore less suited for live cell imaging. However, the R26R-EYFP and the mTmG reporter are less susceptible to basal CreER^T2^ leakage

The effects of Tamoxifen-independent CreER^T2^ activity should be taken into consideration when designing lineage tracing, fate mapping or pulse-chase experiments. Problems can be avoided if a reporter with a relatively high recombination threshold, such as mTmG or R26R-EYFP, is used (Fig. 7) and proper controls are included. However, for different purposes, different lines may be ideal: the Ai14 reporter remains interesting for certain applications due to its brightness and photostability, which makes it superior for live imaging applications (Fig. 7).

## Electronic supplementary material

Supplementary Fig. 1 Activation of fluorescent reporters under basal CreER^T2^ activity levels in the embryonic skin. Left column panels: representative images of the skin of E15 embryos carrying either the Ai14 reporter alone, Cdh5(PAC)-CreER^T2^ and Ai14, or Cdh5(PAC)-CreER^T2^ and mTmG. Basal Cre leakage levels produce abundant recombination in CreER^T2^ Ai14 animals, but very little in CreER^T2^ mTmG. No reporter expressing cells can be detected in the skin of mice carrying only the Ai14 reporter. The vasculature is labeled by CD31 in red, the lymphatics are labeled by Neuropilin2 in blue, and the reporter signal is shown in green. Scale bar indicates 500 μm. Right column panels: high resolution images of the white squares on the left panels. Scale bar indicates 125 μm.

Supplementary material 1 (TIFF 25687 kb)
