# Supplementary figures and images for: Tamoxifen-independent recombination of reporter genes limits lineage tracing and mosaic analysis using CreERT2 lines
Source: Transgenic Res. 2019 Oct 22;29(1):53–68. doi: 10.1007/s11248-019-00177-8 (PMC7000517; doi:10.1007/s11248-019-00177-8)

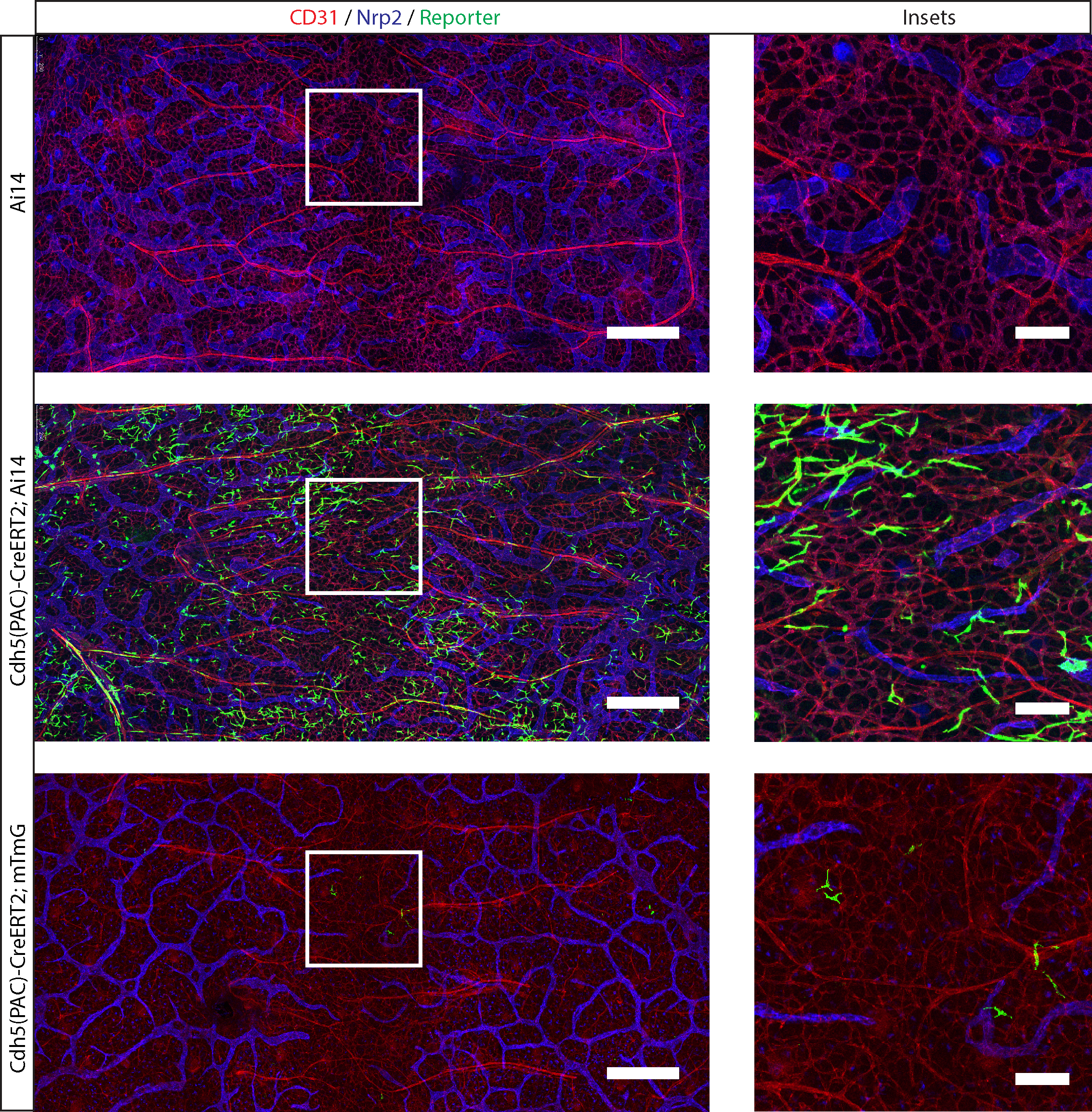

Supplement: Supplementary file 1 — Supplementary material 1 (TIFF 25687 kb) [file 11248_2019_177_MOESM1_ESM.tif]
